# Auricular Acupuncture

**DOI:** 10.1155/2016/4231260

**Published:** 2016-05-18

**Authors:** Gerhard Litscher, Pei-Jing Rong

**Affiliations:** ^1^Research Unit for Complementary and Integrative Laser Medicine, Research Unit of Biomedical Engineering in Anesthesia and Intensive Care Medicine, TCM Research Center Graz, Medical University of Graz, 8036 Graz, Austria; ^2^Institute of Acupuncture and Moxibustion, China Academy of Chinese Medical Sciences, Beijing 100700, China

This special issue includes six interesting manuscripts. Auricular acupuncture is a method which has been successfully used in various fields of medicine. Evidence-based results of this method are of special importance.

This special issue highlights the historical background, the development, and anatomical and neurological aspects of auricular acupuncture and related methods. In detail, the accepted manuscripts deal with the following interesting aspects.

D. H. Iunes et al. investigated the role of auriculotherapy with mustard seeds in the treatment of temporomandibular disorders (TMDs), anxiety, and electromyographic (EMG) activity in university students. The authors used the State Trait Anxiety Inventory (STAI), Research Diagnostic Criteria (RDC) for TMDs (RDC/TMDs), and electromyography in this study of 44 college students with high levels of anxiety and TMDs. The subjects were divided into two groups: an auriculotherapy (AA) group (*n* = 31) and an AA sham group (*n* = 13). The mustard seeds were applied to the shenmen, rim, sympathetic, brain stem, and temporomandibular joint (TMJ) points in the AA group and to sham points in the external ear and wrist in the AA sham group. The treatment protocol consisted of 10 sessions (two treatments per week). The results showed that anxiety (*p* < 0.01) was significantly reduced in the AA group. This group also showed a decrease in tender points in the mandibular posterior region (*p* = 0.04) and in the right side of the submandibular region (*p* = 0.02). Complaints of bilateral pain were reduced in the temporal tendon (*p* ≤ 0.01) and in the left side of the TMJ (*p* < 0.01). In addition, electromyographic (EMG) activity was reduced during temporal muscle contraction (*p* = 0.03). The authors therefore concluded that auriculotherapy was effective in the treatment of students with anxiety and TMDs.

A randomized controlled trial on the effect of auricular acupressure on uremic pruritus in patients receiving hemodialysis treatment was described by C. Yan et al. Uremic pruritus (UP) is a common symptom in patients undergoing maintenance hemodialysis for end-stage renal disease (ESRD). Many nonpharmacological treatments, including acupressure, are currently used to relieve discomfort due to pruritus. The trial was designed to determine the clinical efficacy of auricular acupressure therapy on pruritus in hemodialysis patients and to explore possible underlying mechanisms. Patients receiving maintenance hemodialysis were recruited and assigned to intervention (*n* = 32) and control (*n* = 30) groups. The intervention group underwent auricular acupressure treatment three times a week for six weeks. The patients were asked to press their acupoints 5–8 times daily, with one mandatory press before going to sleep every night. Points on one side of the ear were chosen each time, with alternating bilateral treatment; the tape was replaced every other day and removed every Sunday as a break day. Pruritus scores were assessed using VAS scores, and enzyme-linked immunosorbent assays were used to measure levels of other possible contributory biochemical factors, including calcium, phosphorus, parathyroid hormone (PTH), histamine, substance P, protease activated receptor-2 (PAR-2), and tryptase. Auricular acupressure was not applied to patients in the control group; however, tape without vaccaria seeds was applied to the same six auricular acupoints as the intervention group, and patients were told that the tape contained traditional Chinese medicine that could reduce pruritus. There was a significant difference in mean VAS scores between the postintervention and control groups during follow-up (3.844 ± 1.687 versus 5.567 ± 2.285, *p* < 0.0001). Compared to the control group, serum histamine levels in the postintervention group at the six-week follow-up had decreased significantly (*p* = 0.0290). However, there were no significant differences in serum levels of calcium, phosphorus, PTH, substance P, PAR-2, or tryptase. The findings suggest that auricular acupressure may be a useful treatment in the multidisciplinary management of UP in ESRD patients.

Y. Jiao et al. compared body, auricular, and abdominal acupuncture treatments for insomnia in order to identify the optimum treatment protocol. A three-factor (3 needling protocols) and three-level experimental scheme was designed based on orthogonal method. 54 patients suffering from insomnia, differentiated as internal harassment of phlegm-heat syndrome, were given two courses of acupuncture treatment, each consisting of 20 times of acupuncture. The therapeutic effects were evaluated by comparing the Pittsburgh sleep quality index (PSQI), Hamilton Depression Scale (HAMD) scores, and Hamilton Anxiety Scale (HAMA) scores of patients before treatment, after one course of treatment, after two courses of treatment, and one month after treatment. Body, auricular, and abdominal acupuncture treatments all alleviated symptoms of insomnia, depression, and anxiety, but body and auricular acupuncture had stronger therapeutic effects. The researchers concluded that body acupuncture at basic points should be given priority in protocol selection for insomnia. The second-best choice is auricular acupuncture with basic points combined with points based on traditional Chinese medicine (TCM) theories. Abdominal needling with very quick effects can be an alternative protocol with basic points combined with syndrome differentiation points.

In a review article, P.-W. Hou et al. examined the history, mechanism, and clinical application of auricular therapy in traditional Chinese medicine. Auricular therapy includes acupuncture, electroacupuncture, acupressure, lasering, cauterization, moxibustion, and bloodletting in the auricle. For 2500 years, people have employed auricular therapy for treating diseases, but the methods have been limited to bloodletting and cauterization. Only after 1957 did the international scientific community become aware that the map of the ear resembles an inverted fetus; its introduction has led to auricular acupuncture (AA) becoming a more systemic approach, and following the identification and standardization of more precise points, AA has been employed in clinical applications. The mechanisms of AA are considered to have a close relationship with the autonomic nervous system, the neuroendocrine system, neuroimmunological factors, neuroinflammation, and the neural reflex. The authors found that auricular therapy has been applied, for example, for pain relief, for the treatment of epilepsy, anxiety, and obesity, and for improving sleep quality. In conclusion, however, the mechanisms and evidence for auricular therapy still warrant further study.

An analysis of advantages and disadvantages of the location methods of international auricular acupuncture points (AAPs) was carried out by P.-J. Rong et al. The international standardization of AAPs is an important basis for auricular therapy or auricular diagnosis and treatment. The study on the international standardization of AAPs has gone through a long process, in which the location method is one of the key research projects. There are different points of view in the field of AAPs among experts from different countries or regions. By only analyzing the nine representative location methods, this article tried to offer a proper location method to locate AAPs. Through analysis of the pros and cons of each location method, the location method applied in the WFAS international standard of AAPs is thoroughly considered as an appropriate method by the authors. Yet they also stated that it is important to keep the right direction during developing an ISO international standard of AAPs and to improve the research quality of international standardization for AAPs.

Y. Bian et al. investigated functional connectivity modulation by acupuncture in patients with Bell's palsy (BP), an acute unilateral facial paralysis which is frequently treated with acupuncture in many countries. However, the mechanism of treatment is not clear so far. In order to explore the potential mechanism, 22 healthy volunteers and 17 BP patients with different clinical duration were recruited. The resting-state functional magnetic resonance imaging scans were conducted before and after acupuncture at LI4 (Hegu), respectively. By comparing BP-induced functional connectivity (FC) changes with acupuncture-induced FC changes in the patients, the abnormal increased FC that could be reduced by acupuncture was selected. The FC strength of the selected FC at various stages was analyzed subsequently. The results show that FC modulation of acupuncture is specific and consistent with the tendency of recovery. Therefore, the researchers propose that FC modulation by acupuncture may be beneficial to recovery from the disease.

